# Suppression of the hyaluronic acid pathway induces M1 macrophages polarization via STAT1 in glioblastoma

**DOI:** 10.1038/s41420-022-00973-y

**Published:** 2022-04-11

**Authors:** Tao Yan, Kaikai Wang, Jiafeng Li, Hong Hu, He Yang, Meng Cai, Ruijie Liu, Honglei Li, Ning Wang, Ying Shi, Wei Hua, Huailei Liu

**Affiliations:** 1 Department of Neurosurgery, First Affiliated Hospital of Harbin Medical University, Harbin, China; 2 Key Colleges and Universities Laboratory of Neurosurgery in Heilongjiang Province, Harbin, China; 3 Institute of Neuroscience, Sino-Russian Medical Research Center, Harbin Medical University, Harbin, China; 4 Department of Neurosurgery, The Second Affiliated Hospital, School of Medicine, Zhejiang University, Hangzhou, China; 5 Department of Critical Care Medicine, First Affiliated Hospital of Harbin Medical University, Harbin, China; 6 Department of Radiology, First Affiliated Hospital of Harbin Medical University, Harbin, China; 7 Department of Pathology, First Affiliated Hospital of Harbin Medical University, Harbin, China

**Keywords:** Cancer microenvironment, Cancer microenvironment

## Abstract

Immunosuppressive tumor microenvironment is a crucial factor that impedes the success of tumor immunotherapy, and tumor-associated macrophages (TAMs) are essential for the formation of tumor immunosuppressive microenvironment. Hyaluronic acid (HA) is highly important brick for glioblastoma microenvironment, but whether it contributes to TAM polarization and glioblastoma immunosuppressive microenvironment is less well known. In our study, we observed that disrupting glioblastoma HA synthesis or blocking HA binding to its receptor CD44 on macrophages increased the proportion of M1 macrophages by upregulating SIRPα in macrophages, the underlying mechanism was elevated SIRPα enhanced STAT1 phosphorylation and suppressed STAT3 phosphorylation in macrophages. Subsequently, the induced macrophages could inhibit glioblastoma growth via a feedback effect. In addition, 4-methylumbelliferone (4MU), a cholecystitis drug, can disrupt the CD47/SIRPα axis by disturbing glioblastoma HA synthesis. Collectively, these findings indicated that HA plays a crucial role in macrophages polarization and CD47/SIRPα signaling between glioblastoma cells and macrophages, and suppressing the HA pathway may be a new immunotherapeutic approach for glioblastoma.

## Introduction

Glioblastoma is considered as an incurable and devastating central nervous system tumor, the median survival time is only 15 months [1]. Consequently, new therapeutic approaches for glioblastoma are urgently needed. Immunotherapy has achieved encouraging therapeutic effect in melanoma [2] and non-small cell lung cancer [3], which have rekindled researchers’ faith in cancer treatments. Recently, the unique tumor immunosuppressive microenvironment composed of tumor-associated macrophages (TAMs), myeloid-derived Suppressor Cells (MDSCs), tumor-associated neutrophils (TANs) and related tumor release molecules have been shown to result in unsatisfactory therapeutic outcomes in glioma [4].

To date, immunotherapeutic targets have been identified mainly in the CTLA-4 and PD-1/PD-L1 axis, and related treatments have exhibited antitumor efficacy by activating components of the immune system [5], nevertheless, due to the existence of glioma special microenvironment, the immunotherapy targeting PD-1/PD-L1 is not encouraging in glioma [6]. Recent studies shown that TAMs are closely related to the tumor immunosuppressive microenvironment [7], an increasing number of immunotherapeutic approaches have been aimed at targeting macrophages to treat tumors. TAMs are classified as M1-like TAMs and M2-like TAMs, which have different pathological functions. M1-like TAMs are responsible for the production of proinflammatory cytokines, which are related to the inhibition of tumor progression, M2-like TAMs are correlated with the formation of tumor immunosuppressive microenvironment and tumor progression [8]. TAMs are the predominant infiltrating immune cell population in gliomas, accounting for 30–50% of the total tumor cellular population, which is associated with the poor prognosis and grade of glioma patients [9, 10]. However, the clinical translational potential of regulating macrophage for glioma treatment is still not encouraging. Given that the complex relationship between macrophages and tumors may often be a formidable obstacle to tumor therapeutic outcome [11]. As a crucial phagocytosis signaling pathway between macrophages and tumors, CD47/SIRPα axis is likely to be an effective target for tumor immunotherapy [5]. CD47 and other immunosuppressive molecules are overexpressed in glioblastoma multiforme (GBM) and could bind with their ligands expressed in macrophages, thereby exerting an inhibitory effect on innate and adaptive immune function and ultimately leading to immune escape in GBM [12], meanwhile the elevated expression of CD47 could promote GBM invasion and proliferation [13]. CD47 that is expressed on GBM surface could combine with the V-like domain at the NH2-terminal of SIRPα that is expressed on macrophages result in the phosphorylation of tyrosine residues in the immune-receptor tyrosine-based inhibition motifs (ITIMs), leading to the tyrosine phosphatase SHP1/SHP2’s activation, finally, prevent GBM from phagocytosis by macrophages [5]. Therefore, accumulating evidences indicate that inducing M1-like TAMs polarization and targeting the CD47/SIRPα axis could uncover the clinical translational potential for glioblastoma treatment.

Hyaluronic acid (HA), a major component of the extracellular matrix (ECM), contributes to many physiological processes, including angiogenesis and cancer development [14]. Moreover, many HA signaling pathway molecules are abnormally expressed in cancers [15], therefore, we speculated that targeting the HA pathway could be a therapeutic strategy for glioblastoma. HA influences macrophage/monocyte activation and is associated with an increased number of infiltrating TAMs surrounding tumor cells [16]; for example, abnormal HA accumulation is related to the number and polarization of recruited macrophages in breast cancer [17]. More importantly, HA is an immune system modulator and can regulate the adhesion and migration activities of immune cells that express HA receptors [18]. For instance, HA was found to activated T lymphocytes via CD44 both in vivo and in vitro [19]. Moreover, HA could serve as a barrier in the tumor microenvironment, which limiting accessibility by immune system components [16]. Therefore, inhibiting the HA pathway could interfere with the formation of the tumor immunosuppressive microenvironment. However, whether disrupting the HA synthesis pathway can regulate macrophages polarization and its translational potential in glioblastoma remain unclear and need further study.

Here, we confirmed that interfering with HA synthesis in glioblastoma cells or blocking the binding of HA to CD44 on macrophages induced macrophages polarization toward the M1 phenotype, and the induced macrophages showed a therapeutic effect for glioblastoma. The mechanism underlying this effect was that modulating macrophages polarization toward the M1 phenotype by upregulating STAT1 phosphorylation and downregulating STAT3 phosphorylation. Interestingly, inhibition of glioblastoma HA synthesis with 4-methylumbelliferone (4MU) also led to downregulation of CD47 expression in glioblastoma cells and upregulated SIRPα expression in macrophages, which further facilitated the phagocytosis of glioblastoma cells by macrophages. In summary, considering the clinical safety of 4MU, it could constitute a new strategy for adjuvant chemotherapy in glioblastoma.

## Results

### Inhibiting glioblastoma HA synthesis promotes M1 macrophages polarization

**Fig. 1:**
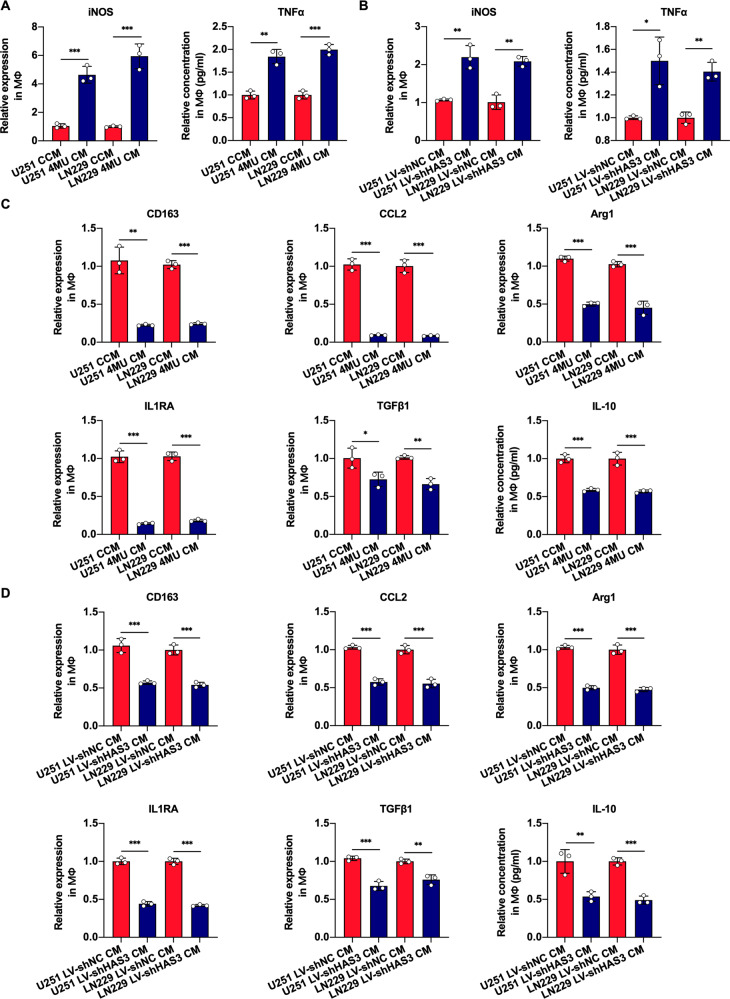
Disruption of HA synthesis in glioblastoma by 4MU treatment or HAS3 knockdown modulates M1 macrophages polarization. **A**–**D** Macrophages were cultured with 4MU CM or LV-shHAS3 CM for 48 h. The relative mRNA expression levels of iNOS, CD163, CCL2, Arg1, IL1RA, and TGFβ1 in macrophages were detected by qRT-PCR. Induced macrophages were cultured alone in FBS-free medium for another 48 h, and the relative concentrations of TNFα and IL-10 secreted from the macrophages were detected by ELISA. Error bars show mean ± SD. *P < 0.05, **P < 0.01, and ***P < 0.001.

In previous study, we confirmed that glioblastoma growth could be inhibited by 4MU treatment or HAS3 silencing via inhibiting HA synthesis [20]. To confirm whether the HA abnormal accumulation in glioblastoma could regulate macrophages polarization, U251 and LN229 cell lines were subjected to 4MU treatment or HAS3 silencing in vitro for 48 h. Then, the culture supernatant was collected and mixed with RPMI-1640 to obtain different conditioned medium (CM). Next, the macrophages were cultured with CM for 48 h to obtain induced macrophages and observe macrophages polarization. Then, we evaluated the mRNA expression levels of M1 macrophages marker (iNOS) and the secretion of TNF-α by induced macrophages using qRT-PCR and ELISA, respectively. The expression levels of iNOS and the secretion of TNF-α were enormously increased in treatment group relative to controls (Fig. 1A, B). Moreover, to gain further insights into the effects of glioblastoma HA synthesis on macrophages polarization, qRT-PCR and ELISA were respectively performed to measure the mRNA expression levels of M2 macrophages marker (CD163, CCL2, Arg1, IL1RA, TGFβ1) and the secretion of IL-10 by induced macrophages. We observed that the expression levels of M2 macrophages marker were significantly decreased, and the IL-10 secreted by macrophages was markedly reduced in treatment group (Fig. 1C, D). To measure the direct effect of 4MU on macrophages polarization, the macrophages were cultured with 4MU for 48 h, then, we used qRT-PCR to detect the expression of M1 (iNOS) and M2 marker (Arg1). The results showed that the expression of iNOS, Arg1 was no statistically significant relative to control group (Supplementary Fig. 1E). Next, we determined the proportion of macrophages expressing CD163 in induced macrophages by flow cytometry. Interestingly, the proportion of CD163^+^ macrophages was decreased in macrophages induced by 4MU CM or LV-shHAS3 CM. Furthermore, exogenous HA rescued the proportion of CD163^+^ macrophages (Supplementary Fig. 1F).

Collectively, these results confirmed that interfering with HA synthesis in glioblastoma could promote M1 macrophages polarization and inhibit M2 macrophages polarization.

### Restricted HA binding to CD44 on macrophages induces M1 macrophages polarization in glioblastoma

**Fig. 2:**
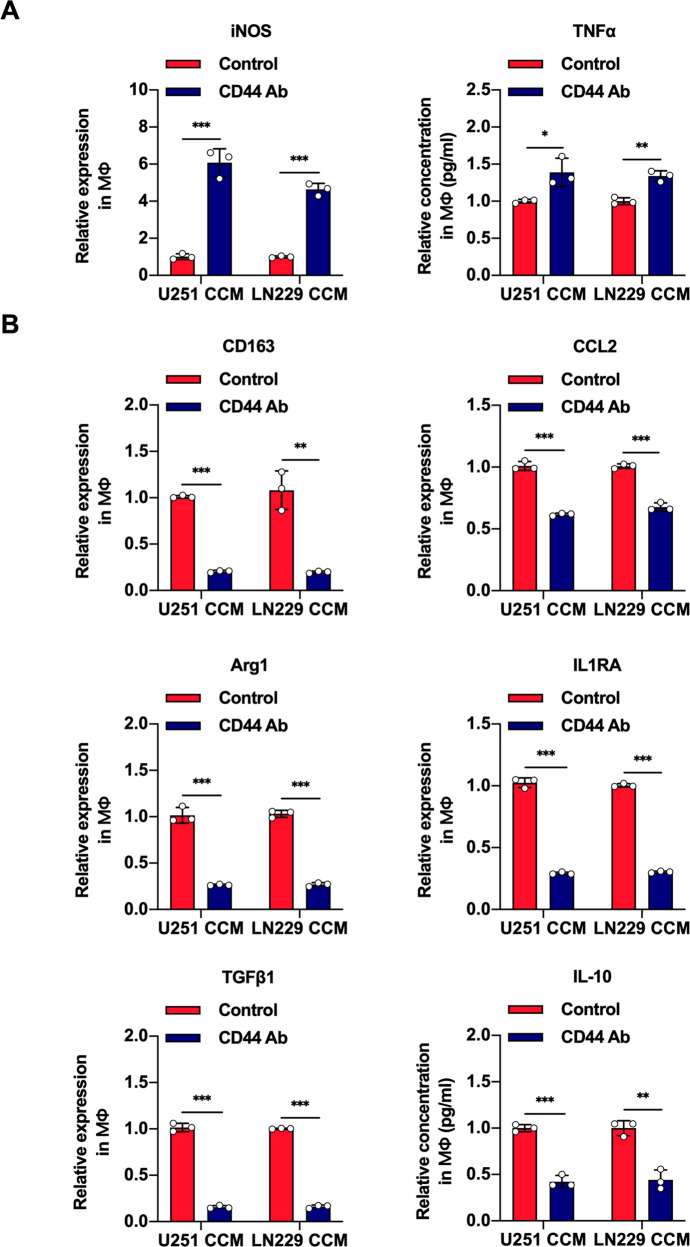
Blocking the binding of HA to CD44 on macrophages modulates M1 macrophages polarization in glioblastoma. **A**, **B** Macrophages pretreated with the anti-CD44 antibody (6 μg/ml) were cultured with CCM for 48 h. The relative mRNA expression levels of iNOS, CD163, CCL2, Arg1, IL1RA, and TGFβ1 in macrophages were detected by qRT-PCR. Induced macrophages were cultured alone in FBS-free medium for another 48 h, and the relative concentrations of TNFα and IL-10 secreted from the macrophages were detected by ELISA. Error bars show mean ± SD. *P < 0.05, **P < 0.01, and ***P < 0.001.

In our previous research, we demonstrated that CD44, as an important HA receptor, binds to HA, which is crucial for glioma progression [20]. Moreover, HA binding to CD44 could regulate T cell activation and tumor progression, and the effect of HA fragment in macrophages could be partially inhibited by anti-CD44 antibodies [21]. Therefore, in our present study, we explored whether the effect of HA on macrophages polarization may be mediated via CD44. First, macrophages were pretreated with anti-CD44 antibody and then cultured with CCM. qRT-PCR and ELISA were performed to detect the expression of iNOS and the relative contents of TNF-α secreted by macrophages. The results showed that anti-CD44 antibody pretreatment led to increased expression of iNOS and elevated secretion of TNF-α in macrophages (Fig. 2A). Next, we determined the expression of CD163, CCL2, Arg1, IL1RA, TGFβ1, and the secretion of IL-10 in macrophages. As expected, the expression of CD163, CCL2, Arg1, IL1RA, and TGFβ1 and the production of IL-10 were reduced in macrophages pretreated with anti-CD44 antibody (Fig. 2B). Furthermore, we measured the proportion of macrophages expressing CD163 by flow cytometry. Blockade of CD44 on macrophage surface reduced the proportion of macrophages expressing CD163. Moreover, additional HA partially rescued the inhibitory outcome of anti-CD44 antibody pretreatment (Supplementary Fig. 1G). Taken together, these findings indicated that blocking the binding of HA to CD44 promotes M1 macrophages polarization.

### Induced macrophages are associated with a positive therapeutic outcome of glioblastoma in vitro and in vivo

**Fig. 3:**
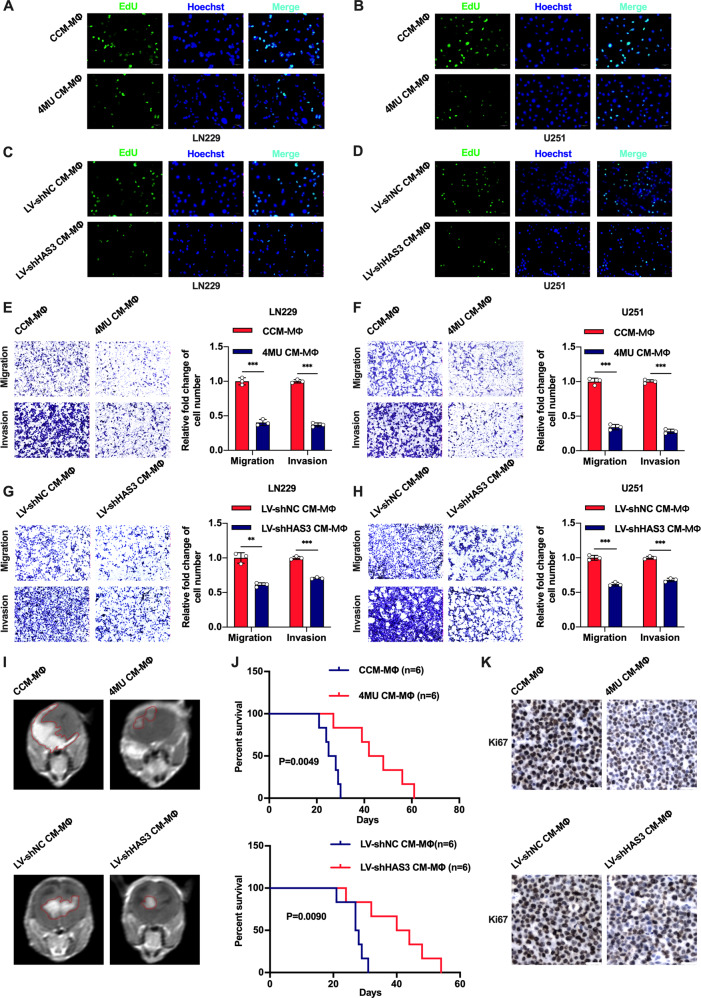
Macrophages induced by 4MU CM or LV-shHAS3 CM inhibit glioblastoma cell proliferation and motility in vitro and in vivo. **A**–**H** Macrophages were cultured with 4MU CM or LV-shHAS3 CM for 48 h. Then, induced macrophages were subsequently cocultured with U251 or LN229 glioblastoma cells for 48 h. An EdU assay was performed to test the glioblastoma cells proliferation (bar: 50 μm). Transwell assays were used to analyze the glioblastoma cells migration and invasion abilities (bar: 100 μm). **I**–**J** Representative MRI scans and survival curves of xenograft tumors in mice co-implanted with glioblastoma cells and induced macrophages. Four xenograft model groups were established, as follows: (1) glioblastoma cells and CCM-derived macrophages (Median survival time: 26.5 days), (2) glioblastoma cells and 4MU CM-derived macrophages (Median survival time: 45 days), (3) glioblastoma cells and LV-shNC CM-derived macrophages (Median survival time: 27.5 days), (4) glioblastoma cells and LV-shHAS3 CM-derived macrophages (Median survival time: 42 days). **K** Representative images of Ki67 IHC staining in xenograft glioblastoma tissues are described in Fig. 3I. (bar: 30 µm) Error bars shown mean ± SD. *P < 0.05, **P < 0.01, and ***P < 0.001.

According to our results, we considered that disruption of glioblastoma HA synthesis or blocking the binding of HA to CD44 on macrophages could modulated the phenotype of macrophages. Thus, we inferred that the polarization of macrophages might exert effects on the malignant biological behavior of glioblastoma. To verify this hypothesis, macrophages were cultured with 4MU-derived CM or LV-shHAS3-derived CM to generate induced macrophages, and then the obtained macrophages were cocultured with glioblastoma cells. The subsequent EdU assay revealed that induced macrophages significantly inhibited glioblastoma proliferation (Fig. 3A–D, Supplementary Fig. 1H, I). In addition, Transwell assays showed that induced macrophages reduced glioblastoma cells mobility (Fig. 3E–H). To further clarify this feedback effect, the induced macrophages were co-implanted with LN229 cell line into nude mice brain. Similar to the in vitro results, the glioblastoma growth was suppressed and the survival time was extended relative to control group mice in vivo. Moreover, compared with controls, experimental group mice exhibited decreased expression of Ki67, a proliferation marker, in glioblastoma tissues (Fig. 3I–K).

**Fig. 4:**
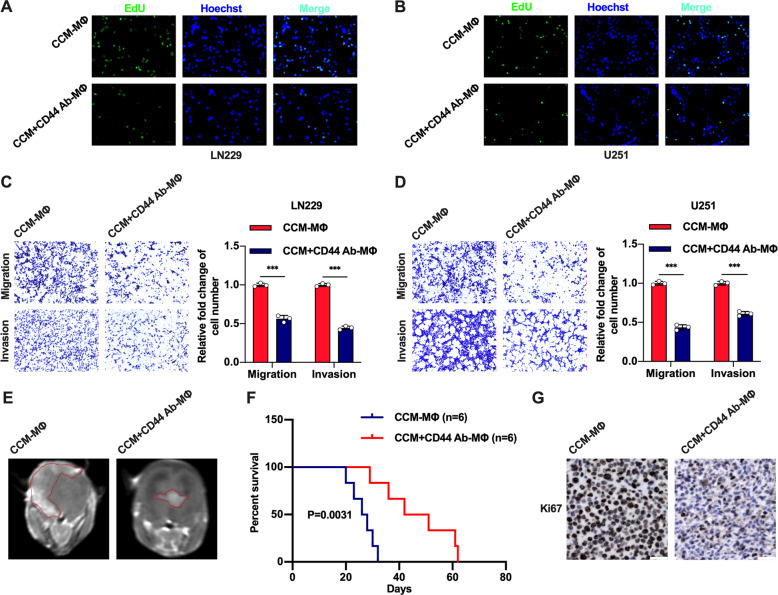
Macrophages induced by anti-CD44 antibody and CCM inhibit glioblastoma cell proliferation and motility in vitro and in vivo. **A**–**D** Macrophages pretreated with the anti-CD44 antibody (6 μg/ml) were cultured with CCM for 48 h. Then, induced macrophages were subsequently cocultured with U251 or LN229 glioblastoma cells for 48 h. An EdU assay was performed to test the glioblastoma cells proliferation (bar: 50 μm). Transwell assays were used to analyze the glioblastoma cells migration and invasion abilities. (bar: 100 µm) **E**, **F** Representative MRI scans and survival curves of xenograft tumors from mice co-implanted with glioblastoma cells and macrophages. Two xenograft model groups were established, as follows: (1) glioblastoma cells and CCM-derived macrophages (Median survival time: 27 days), (2) glioblastoma cells and CCM-derived macrophages pretreated with anti-CD44 antibody (Median survival time: 46.5 days). **G** Representative images of Ki67 IHC staining in xenograft tumor tissues are described in Fig. 4E (bar: 30 μm). Error bars show mean ± SD. *P < 0.05, **P < 0.01, and ***P < 0.001.

Moreover, we investigated the effect of macrophages pretreated with anti-CD44 antibody on glioblastoma cells. The macrophages were pretreated with anti-CD44 antibody and then cultured with CCM for 48 h to obtain induced macrophages. Then induced macrophages were cocultured with U251 and LN229 cell lines. Subsequently, EdU assay revealed that the glioblastoma proliferation was markedly decreased (Fig. 4A, B, Supplementary Fig. 1J), and the Transwell assay showed that the glioblastoma cells migration and invasion were significantly reduced (Fig. 4C, D). To further evaluate these effects in vivo, another glioblastoma-bearing mouse model was established, in which induced macrophages were co-implanted with LN229 cell line. The survival time of experimental mice was more prolonged than that in control group, and glioblastoma growth was significantly suppressed (Fig. 4E, F). Immunohistochemical (IHC) staining showed that the expression level of Ki67 in glioblastoma tissue was markedly reduced in experimental mice (Fig. 4G).

Overall, these data supported the conclusion that induced macrophages have a feedback-mediated inhibitory effect on glioblastoma growth.

### Interfering HA synthesis in glioblastoma or disrupting the binding of HA to CD44 on macrophages promotes M1 macrophages polarization via STAT1 activation

**Fig. 5:**
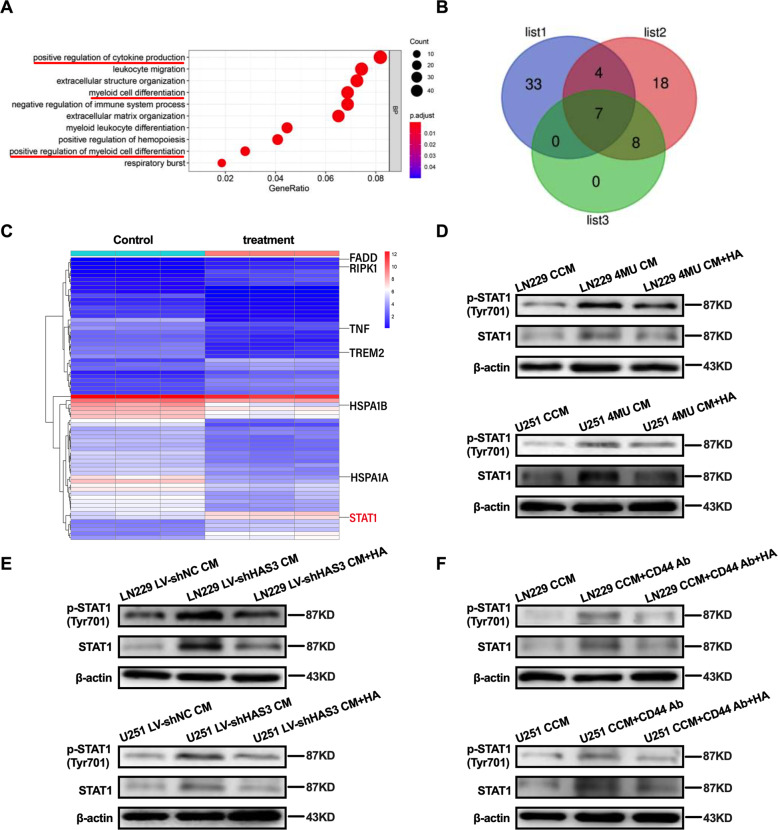
Interfering HA synthesis in glioblastoma or disrupting the binding of HA to CD44 on macrophages promotes M1 macrophages polarization via STAT1 activation. **A** Differentially expressed genes were subjected to GO analysis, and the results showed that these differentially expressed genes were highly associated with the following three biological processes: positive regulation of cytokine production, myeloid cell differentiation and positive regulation of myeloid cell differentiation. **B** The differentially expressed genes common to the above three biological processes were further analyzed by a Venn diagram, and 7 overlapping genes were identified. (list1: positive regulation of cytokine production; list2: myeloid cell differentiation; list3: positive regulation of myeloid cell differentiation) **C** Heat map of the differentially expressed genes involved in the above three biological processes; the red mark indicates STAT1. **D** Macrophages were cultured with 4MU CM for 48 h, and one of the treatment groups was extra treated with exogenous HA (25 μg/ml). The relative protein levels of p-STAT1 and STAT1 in macrophages were examined by Western blotting. **E** Macrophages were cultured with LV-shHAS3 CM for 48 h, and one of the treatment groups was extra treated with exogenous HA (25 μg/ml). The relative protein levels of p-STAT1 and STAT1 in macrophages were examined by Western blotting. **F** Macrophages pretreated with the anti-CD44 antibody (6 μg/ml) were cultured with CCM for 48 h, one of the treatment groups was extra treated with exogenous HA(25 μg/ml). The relative protein levels of p-STAT1 and STAT1 in macrophages were determined by Western blotting. Error bars show mean ± SD. *P < 0.05, **P < 0.01, and ***P < 0.001.

Given that interfering with HA pathway could regulate macrophages polarization, which prompted us to explore its potential mechanisms. Firstly, Glioblastoma cells were treated with 4MU for 48 h to obtain 4MU-derived CM, and then, macrophages were cultured with 4MU-derived CM for another 48 h to generate induced macrophages. Next, the mRNA sequencing analysis was performed to analyze the expression of genes in induced macrophages. A total of 753 genes were found to be differentially expressed (p < 0.05, |log2FC | ≥1). Through gene ontology (GO) bioinformatic analysis, biological processes (BPs) such as positive regulation of cytokine production, myeloid cell differentiation and positive regulation of myeloid cell differentiation were markedly differentially regulated when macrophages were cultured with 4MU-derived CM (Fig. 5A). Next, the intersection of these genes was determined via a Venn diagram, and 7 overlapping genes were ultimately identified: TREM2, TNF, HSPA1A, HSPA1B, RIPK1, FADD, and STAT1 (Fig. 5B, C). Interestingly, STAT1 activation is an important driver of M1 macrophages polarization [22], therefore, in our research, we speculated that STAT1 may be related to the transformation of macrophage phenotype. To test this hypothesis, we evaluated the protein level of p-STAT1 (Tyr701) in macrophages and observed that it was markedly increased in macrophages when macrophages were cultured with 4MU CM or LV-shHAS3 CM (Fig. 5D, E). Moreover, the level of p-STAT1 was enhanced when anti-CD44 pretreated macrophages were cultured with CCM (Fig. 5F). And, exogenous HA reversed the above phenomenons (Fig. 5D–F). In addition, STAT3 activation is also a key determinant of M2 macrophages polarization [23]. For example, under hypoxic conditions, glioma-derived exosomes can induce M2 macrophages polarization via the STAT3 pathway [24]. Therefore, we hypothesized that STAT3 might be associated with macrophages polarization observed in our study. Indeed, we observed that STAT3 (Tyr705) phosphorylation was impaired in macrophages, after macrophages were cultured with 4MU CM or LV-shHAS3 CM. And the level of p-STAT3 in macrophages was reduced when macrophages pretreated with the anti-CD44 antibody were cultured with CCM for 48 h. Moreover, this reduction was reversed by treatment with exogenous HA (Supplementary Fig. 1K–M). Collectively, these observations indicated that interfering with the HA pathway could promote M1 macrophages polarization via activation of STAT1 phosphorylation and inhibited M2 macrophages polarization via impairment of STAT3 phosphorylation in glioblastoma.

### 4MU disrupts the CD47-SIRPα axis between glioblastoma and macrophages

**Fig. 6:**
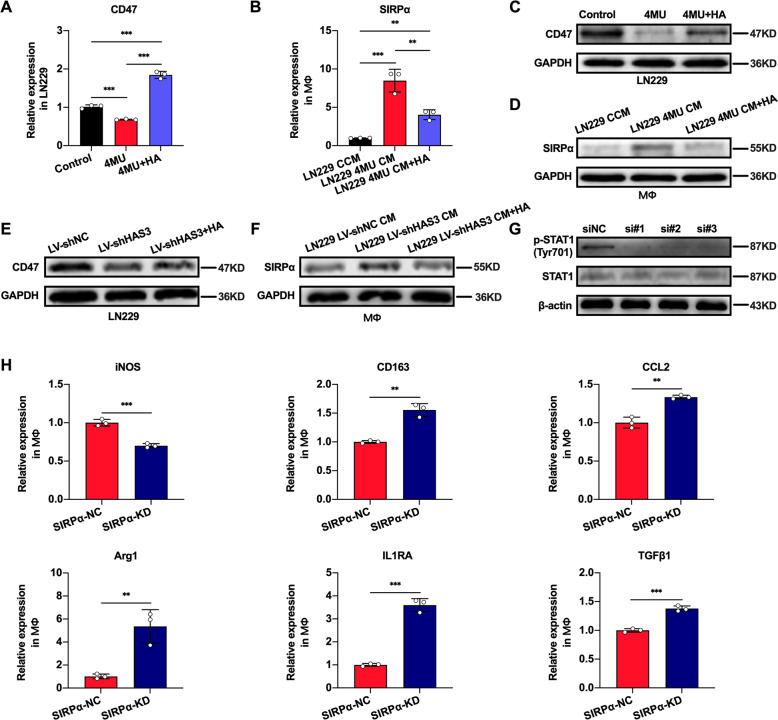
4MU interferes with CD47-SIRPα signaling between glioblastoma cells and macrophages. **A**, **C** Glioblastoma cells were cultured with 4MU (1 mmol/L) or 4MU (1 mmol/L) combined with HA (25 μg/ml) for 48 h. The relative levels of CD47 mRNA and protein in glioblastoma were examined by qRT-PCR and Western blotting, respectively. **B**, **D** Macrophages were cultured with 4MU CM for 48 h, and one of the treatment groups was extra treated with exogenous HA (25 μg/ml). The relative expression levels of SIRPα mRNA and protein in macrophages were determined by qRT-PCR and Western blotting, respectively. **E** The relative levels of CD47 protein in LN229 glioblastoma cells stably transfected with HAS3 knockdown lentivirus, followed by cultured with HA (25 μg/ml) for 48 h. **F** Macrophages were cultured with LV-shHAS3 CM for 48 h, and one of the treatment groups was extra-treated with exogenous HA (25 μg/ml). The relative protein levels of SIRPα in macrophages were examined by Western blotting. **G** The relative protein levels of p-STAT1, STAT1 in macrophages with SIRPα knockdown were determined by Western blotting. **H** The relative mRNA expression levels of iNOS, CD163, CCL2, Arg1, IL1RA, and TGFβ1 were detected by qRT-PCR, after SIRPα was knocked down in macrophages. Error bars show mean ± SD. *P < 0.05, **P < 0.01, and ***P < 0.001.

**Fig. 7:**
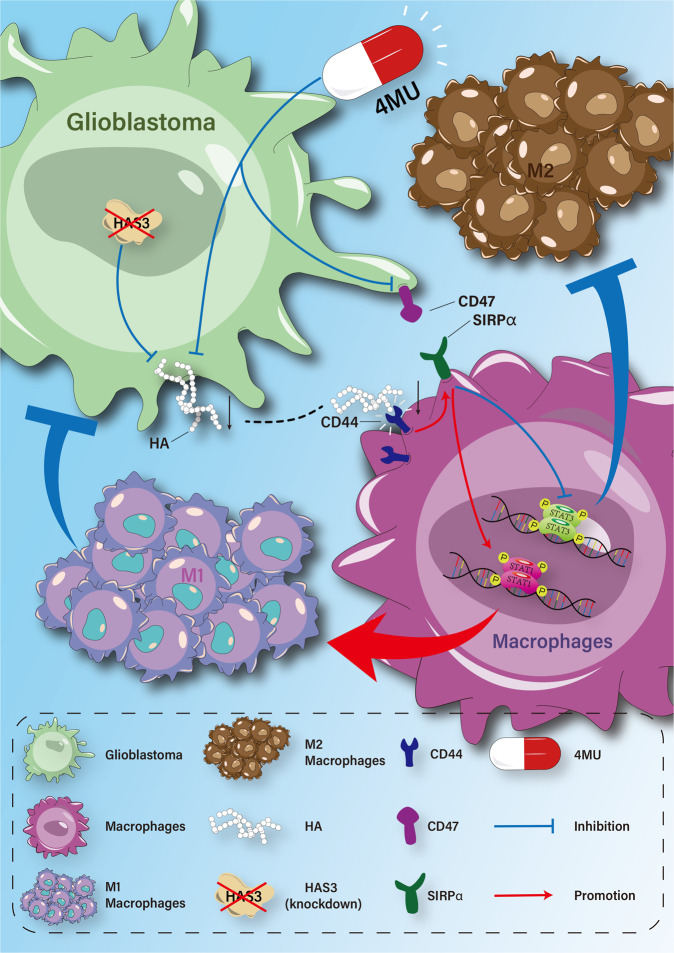
Schematic illustration of the communication between glioblastoma cells and macrophages with disruption of HA pathway in glioblastoma and macrophages coculture conditions.

Clinically, 4MU is often used to relieve biliary spasm [25]. Moreover, we confirmed that 4MU as HA inhibitor could suppress glioma growth in previous research [20]. Meanwhile, CD47 expression in hepatic cancer stem cells can be inhibited by 4MU [26], and CD47-SIRPα axis exerts an inhibitory effect on the ability of macrophages to phagocytose cancer cells [27]. Thus, the role of 4MU for CD47-SIRPα axis in our study was further explored. qRT-PCR and Western blotting were adopted to examine CD47 expression in glioblastoma cells treated with 4MU. Our observations confirmed that after glioblastoma cells were treated with 4MU for 48 h, the expression level of CD47 was significantly reduced, and this inhibitory effect was reversed by exogenous HA (Fig. 6A, C). Given the crucial role of the CD47-SIRPα axis in tumor progression, we next evaluated the expression level of SIRPα in macrophages cultured with 4MU CM. Interestingly, the expression level of SIRPα in macrophages induced by 4MU CM was enhanced. In addition, treatment with exogenous HA reversed the increased SIRPα expression in macrophages (Fig. 6B, D). Moreover, HAS3 knockdown in glioblastoma has the similar effects as 4MU, we observed that the expression of CD47 in glioblastoma could be suppressed by HAS3 knockdown and the expression level of SIRPα in macrophages induced by LV-shHAS3 CM was increased, in addition, these effects could be rescued by exogenous HA (Fig. 6E, F). These results suggested that 4MU could affect the CD47-SIRPα axis between glioblastomas and macrophages by disrupting glioblastoma HA synthesis. We further determined the potential mechanism by which increased SIRPα expression in macrophages for glioblastoma progression in our study. We studied the effect of SIRPα knockdown (SIRPα KD) in macrophages and found that the level of p-STAT1 was decreased, and p-STAT3 was increased in SIRPα KD macrophages (Fig. 6G, Supplementary Fig. 1N, O). Moreover, SIRPα knockdown (SIRPα KD) in macrophages led to decreased expression of M1 macrophages marker (iNOS), the expression of M2 macrophages marker (CD163, CCL2, Arg1, IL1RA and TGFβ1) was increased (Fig. 6H). These data further supported that 4MU could exert an inhibitory effect on CD47 expression in glioblastoma cells and increase SIRPα expression in induced macrophages, further enhancing STAT1 phosphorylation and finally promoting M1 macrophages polarization (Fig. 7).

## Discussion

In the tumor immunosuppressive microenvironment, TAMs play a crucial role in promoting the malignant tumor phenotype. Thus, modulating TAMs polarization is a feasible approach to improve the effects of immunotherapy in glioma. HA is closely associated with the tumor microenvironment [28], and our previous studies demonstrated that blocking the HA pathway can inhibit glioma growth [20]. However, whether the HA pathway influences TAMs remains to be further clarified in glioma. In this study, our results revealed that interfering with HA synthesis in glioblastoma or inhibiting the binding of HA to CD44 on macrophages inhibits M2 macrophages polarization and activates M1 macrophages polarization by regulating STAT3 and STAT1 phosphorylation.

Abnormal accumulation of HA mediates tumor progression [14]. In the presence of three key enzymes (HAS1, HAS2, and HAS3), HA synthesis from the precursors UDP-N-acetylglucosamine and UDP-glucuronic acid is initiated [29]. HA can be classified as high molecular weight HA (HMW-HA), low molecular weight HA (LMW-HA), and oligomeric HA (oHA) according to molecular mass. These types of HA have different functions in tumors. HMW-HA inhibits inflammation and suppresses tumor malignancy, LMW-HA promotes inflammation and tumor progression, and oHA is related to the promotion of wound healing and elimination of therapeutic resistance in tumors [25]. CD44 acts as the primary HA receptor and is widely expressed on the cell membrane surface. Regarding pathological processes in tumors, the binding of HA to CD44 can regulate immune cell recruitment, T cell activation, immune response, and tumor progression [21, 30]. In addition, CD44 participates in the degradation of HA. In the presence of CD44, HMW-HA can be degraded to LMW-HA and then perform its physiological function [14]. In our previous research, we confirmed that HA pathway plays an essential role in glioma progression [20]. Based on the above evidence, we sought to determine whether disturbing HA pathway participates in macrophages polarization in glioblastoma. We found that inhibiting HA synthesis in glioblastoma cells by 4MU treatment or HAS3 silencing regulated macrophages polarization and increased the proportion of M1 macrophages, and that exogenous HA reversed the proportion of M1 macrophages among total macrophages. In addition, we found similar effect when CD44 in macrophages was blocked, and exogenous HA partially reversed these effects induced by CD44 blocked. Our result indicated that the effect of HA on macrophages is partially mediated by CD44, but whether the biological role of HA on macrophages could be regulated by other receptors remains to be explored. Therefore, these data indicated that HA abnormal accumulation in glioblastoma plays a vital role in the M1/M2 phenotypic transformation of macrophages. In our present study, we mainly research the role of HA pathway in glioblastoma on macrophages polarization. However, we did not discuss the effect of macrophage HA metabolism on itself in the present study, which is also a topic in our future research. At present, the construction of macrophage model using U937 cells is considered to be a credible method [24, 31, 32], but it’s not exactly the same as macrophages in glioma microenviroment, which is also a problem in the current research in this field.

TAMs infiltration is a crucial feature in glioma immune-microenvironment and is closely related to the prognosis of glioma patients [9]. In the ECM, an increasing number of molecules are being found to be associated with macrophage activation. Among these molecules, HA and its receptor, CD44, have been demonstrated to regulate immune responses [30]. For instance, LMW-HA can increase IL-8 expression in human melanoma cells via the Toll-like receptor (TLR) 4 and NF-κB pathways [33]. Some studies have reported that abnormal HA accumulation is correlated with an increased number of M2 macrophages via activation of the ERK1/2-STAT3 pathway in breast cancer [34]. Other researchers also confirmed that the binding of HA to CD44 can promote the upregulation of M2 polarization-related genes in human THP-1 cells via the STAT3 pathway [35]. Tyrosine phosphorylation and dimerization of STAT1 can be modulated by JAK, which plays a fundamental role in binding to the DNA of various M1 activation-related genes (e.g., iNOS, MHCII, and IL-12) [36]. STAT1 also regulates SOCS3 expression, which decreases STAT3 activation [37]. To explore the underlying mechanisms in our study, mRNA sequencing analysis was used to capture the potential genes. The result confirmed that the mRNA expression of STAT1 was upregulated. And Western blotting showed that the protein level of p-STAT1 was increased. Collectively, these indirect and direct evidences further confirmed that interfering with glioblastoma HA pathway promotes M1 macrophages polarization through STAT1 activation.

CD47, an inhibitory ligand expressed on tumor cells, often interacts with SIRPα on macrophages to transmit a “don’t eat me” signal to escape the phagocytosis by macrophages. Therefore, targeting the CD47/SIRPα axis seems to exhibit very promising efficacy for tumor therapy, which may unlock the therapeutic potential of macrophages [5, 38]. Given that 4MU can reduce the expression of CD47 on hepatic cancer stem cells [26], we sought to determine whether a similar effect could be seen in glioblastoma. By inhibiting HA synthesis in glioblastoma cells with 4MU, we found that the expression level of CD47 was decreased in glioblastoma cells and that SIRPα expression was increased in macrophages. The increased expression of SIRPα in macrophages could subsequently result in enhanced STAT1 phosphorylation and positively affect M1 macrophages polarization, the induced M1 macrophages contribute to the phagocytosis of glioblastoma cells. The elevated levels of SIPRα seem to contradict the “don’t eat me” signal from the CD47/SIPRα axis. This phenomenon could be explained as follows: to protect glioblastoma cells from phagocytosis by macrophages, the reduced expression of CD47 on the glioblastoma cell surface can lead to increased expression of SIRPα in macrophages via a feedback mechanism. However, macrophages are not entirely controlled by glioblastoma cells, and upregulated expression of SIPRα enhances M1 macrophages polarization through activation of STAT1 phosphorylation via an underlying protective mechanism. This result is also consistent with previous research, which indicated that SIRPα knockdown in macrophages led to enhanced STAT3 phosphorylation and suppressed STAT1 phosphorylation in hepatocellular carcinoma [39]. In our future research, we will focus on this potential protective mechanism in macrophages.

In summary, we found that inhibiting the HA pathway in glioblastoma cells can increase the M1/M2 macrophages ratio. Mechanistically, abnormal HA accumulation could bind to CD44 receptor on macrophages surface, which could regulate downstream STAT1 and STAT3 pathways in macrophages, further leading to the glioblastoma immunosuppressive microenvironment formation and ultimately promoting glioblastoma progression. 4 MU, a small molecule inhibitor of HA, could have specific features: 1. the ability to inhibit the synthesis of HA [25]; 2. the ability to cross the blood–brain barrier (BBB) [40]; 3. inhibition of CD47 expression in glioblastoma cells; 4. safety for oral administration [41]. Therefore, 4MU may be a potential drug for glioblastoma treatment. Finally, our study provides new knowledge of the glioblastoma immunosuppressive microenvironment that will be helpful for guiding glioblastoma immunotherapy.

## Materials and methods

### Cell culture and reagents

Human U937 monocytes and glioblastoma cell lines (U251 and LN229) were derived from the China Infrastructure of Cell Line Resource (National Science & Technology Infrastructure, NSTI). Dulbecco’s modified Eagle’s medium (DMEM; D6429, Sigma, USA) with 10% fetal bovine serum (FBS; 16000-044, Gibco, USA) was used to culture glioblastoma cells, and U937 cells were cultured with Roswell Park Memorial Institute (RPMI)-1640 medium (RPMI-1640; R8758, Sigma, USA) containing 10% FBS (16000-044, Gibco, USA). Human U937 monocytes were induced to differentiate into macrophages (MΦ) with PMA (Cat#HY-18739, MCE, USA) for 24 h in vitro [24], and then macrophages (MΦ) was cultured with different conditioned medium to obtain induced macrophages (Supplementary Fig. 1B, C). The above cells were placed in a 37 °C incubator with 5% CO_2_. 4MU (Cat#M1381, USA) was obtained from Sigma-Aldrich and CD44 antibody (Cat#217594, USA) was derived from Millipore. Hyaluronic acid sodium (Cat#HY-B0633, USA) was purchased from MedChemExpress.

### Conditioned medium (CM) preparation

Glioblastoma cell lines were treated without or with 4MU (1 mmol/L) for 48 h. Next, the culture supernatant was collected. Finally, the collected culture supernatant was mixed with RPMI-1640 in a ratio of 1:1 to obtain control conditioned medium (CCM) and 4MU-derived conditioned medium (4MU CM). Glioblastoma cell lines stably transfected with NC/HAS3-knockdown lentivirus were cultured for 48 h. Next, the culture supernatant was collected and mixed with RPMI-1640 in a ratio of 1:1 to obtain LV-shNC-derived conditioned medium (LV-shNC CM) and LV-shHAS3-derived conditioned medium (LV-shHAS3 CM). The simplified process of conditioned medium preparation were shown in Supplementary Fig. 1A.

### Cell transfection

Macrophages were transfected with siRNA by Lipofectamine 2000 reagent (Cat# 11668019, Invitrogen, USA). SiRNAs (siSIRPα) were obtained from General Biosystems (China). Glioblastoma cell lines were transfected with Lentiviruses (shNC and shHAS3) to obtain stable glioblastoma cell lines, and Lentiviruses (shNC and shHAS3) were derived from Wanleibio (China). The SiRNAs (siSIRPα) and Lentiviruses (shNC and shHAS3) sequences are as follows in Table 1. (LV-shNC: negative control lentivirus, LV-shHAS3: HAS3-knockdown lentivirus).

**Table 1:** Sequence of siRNA, primer, and lentivirus.

siRNA sequence	
siNC	5′UUCUCCGAACGUGUCACGUTT3′
SIRPα-si#1	5′GGUUGCAGCUGGAGAGACATT3′
SIRPα-si#2	5′GAAGAAUGCCAGAGAAAUATT3′
SIRPα-si#3	5′CCGAUGACGUGGAGUUUAATT3′
Primer sequence	
GAPDH	F-5′ CACCCACTCCTCCACCTTTGA3′, R-5′ACCACCCTGTTGCTGTAGCCA3′
CD163	F-5′TTTGTCAACTTGAGTCCCTTCAC3′, R-5′TCCCGCTACACTTGTTTTCAC3′
CCL2	F-5′CAGCCAGATGCAATCAATGCC3′, R-5′TGGAATCCTGAACCCACTTCT3′
IL1RA	F-5′CATTGAGCCTCATGCTCTGTT3′, R-5′CGCTGTCTGAGCGGATGAA3′
iNOS	F-5′TTCAGTATCACAACCTCAGCAAG3′, R-5′TGGACCTGCAAGTTAAAATCCC3′
TGFβ1	F-5′GGCCAGATCCTGTCCAAGC3′, R-5′GTGGGTTTCCACCATTAGCAC3′
Arg1	F-5′GTGGAAACTTGCATGGACAAC3′, R-5′AATCCTGGCACATCGGGAATC3′
CD14	F-5′ACGCCAGAACCTTGTGAGC3′, R-5′GCATGGATCTCCACCTCTACTG3′
CD68	F-5′GGAAATGCCACGGTTCATCCA3′, R-5′TGGGGTTCAGTACAGAGATGC3′
SIRPα	F-5′GGCCTCAACCGTTACAGAGAA3′, R-5′GTTCCGTTCATTAGATCCAGTGT3′
Lentivirus sequences	
LV-shNC	5′GUA UGA CAA CAG CCU CAA GTT3′
LV-shHAS3	5′GGC UAC CGA ACU AAG UAU ATT3′

### qRT-PCR

TRIzol reagent (Cat#T9424, Sigma, USA) was used to extract Total RNA. A Roche Transcriptor cDNA Synthesis Kit (Cat#4897030001, Roche, Switerland) was used for reverse transcription. The expression of the target genes was evaluated with a SYBR Green PCR Master Mix Kit (Cat#4913914001, Roche, Switerland). ABI Prism 7500 rapid thermocycler (Applied Biosystems, USA) was utilized to carry out the experiment of qRT-PCR. The target genes primer sequences are as follows in Table 1.

### Flow cytometry

Macrophages were stained with anti-CD163-PE (Cat#PE-65169, Proteintech) and anti-CD11b-APC (Cat#APC-65116, Proteintech) antibodies at room temperature for 15 min. The proportion of CD11b^+^ CD163^+^ macrophages was detected using flow cytometry.

### ELISA

Macrophage-derived supernatant was collected to measure the secretion of IL-10 (Cat#KE00170, Proteintech, USA) and TNF-α (Cat#KE00154, Proteintech, USA) with ELISA kits. Finally, BioTek ELx800 (USA) microplate reader was used to measure the optical density (OD).

### Glioblastoma cell migration and invasion assays

Induced macrophages were cocultured with glioblastoma cells in a Transwell chamber for 48 h. Induced macrophages were placed in the lower chamber and glioblastoma cells were placed in the upper chamber. After coculture, glioblastoma cells were detached from the upper chamber. The glioblastoma invasion and migration abilities were assessed by Transwell chambers (Cat#TCS003024: JET BIOFIL, China) with or without Matrigel (Cat#356234, Corning, USA). A schematic diagram of the specific method was shown in Supplementary Fig. 1D.

### Glioblastoma cell proliferation assay

Induced macrophages were cocultured with glioblastoma cells (LN229 and U251) in a Transwell chamber for 48 h. Induced macrophages were placed in the upper chamber and glioblastoma cells were placed in the lower chamber (Schematic diagram was shown in Supplementary Fig. 1D). After coculture, glioblastoma cells were obtained from the bottom chamber. An EdU assay kit (Beyotime, China) was used to determine the glioblastoma cells proliferation ability according to the manufacturer’s protocol.

### mRNA sequencing analysis

Induced macrophages mRNA was sequenced using the Illumina sequencing platform. The expression data were expressed as normalized FPKM values, and the differentially expressed genes were identified with the limma package in R software using the default values. Differentially expressed genes were defined as those with |log2FC | ≥ 1 and adjusted *p*-value < 0.05. The differentially expressed genes were subjected to GO analysis with specific R packages. The Venn Diagram was obtained from https://bioinformatics.psb.ugent.be/webtools/Venn/. The R packages were downloaded from http://bioconductor.org/.

### Western blot analysis

Protein samples were obtained from macrophages and glioblastoma cells. The processes of Western blot were consistent with previously reported [20]. Immunoreactions were visualized with a GeneGnome XRQ Imaging System (Syngene, UK). Primary antibodies utilized in our research were as follows: STAT3 (AF6294, Affinity), p-STAT3(YP0251, Immunoway), STAT1 (YT4439, Immunoway), p-STAT1 (YP0249, Immunoway), SIRPα (YT4301, Immunoway), CD47 (YT5509, Immunoway), β-actin (TA-09, ZSGB-BIO), and GAPDH (BA2913, BOSTER).

### Immunohistochemical (IHC) staining

Glioblastoma tissues were derived from orthotopic xenograft model. The obtained sections were utilized for IHC staining as previously reported [20]. Ki67 antibody (A2094, Clone AB) was used in IHC staining.

### Animal model

The nude mice were derived from Beijing Vital River Experimental Animal Technology Co., Ltd. (China). Glioblastoma cells (1 × 10^6^) and induced macrophages (1 × 10^6^) in serum-free DMEM (7 µl) were co-implanted into the brain tissue of the mice. The injection site was located at the midline right 2.5 mm, 0.5 mm posterior to the coronal suture, 3.5 mm below the skull [20]. Three weeks later, MRI scans were used to assess tumor size. Then, the mice were fed until death. Based on the lifetime of mice with glioblastoma, the mice survival curve was drawn. After the glioblastoma-bearing mouse model was established, the mouse breeder was blinded to the group allocation during the experiment. The research ethics were allowed by the Ethics Committee of the First Affiliated Hospital of Harbin Medical University and accorded with the principles of the Declaration of Helsinki.

### Statistical analysis

Student’s *t*-test or one-way analysis of variance (Prism software version 8.0) was used for comparisons of data between groups. Values of *p* < 0.05, <0.01, and <0.001 are represented by different numbers of asterisks in the graphs.

## Supplementary information

Original western blots

Supplementray Figure

Supplementary Figure Legend

## Data Availability

The data generated or analyzed during this study are included in this published article and its supplementary information files.
