# Supplementary figures and images for: Suppression of the hyaluronic acid pathway induces M1 macrophages polarization via STAT1 in glioblastoma
Source: Cell Death Discov. 2022 Apr 11;8:193. doi: 10.1038/s41420-022-00973-y (PMC9001679; doi:10.1038/s41420-022-00973-y)

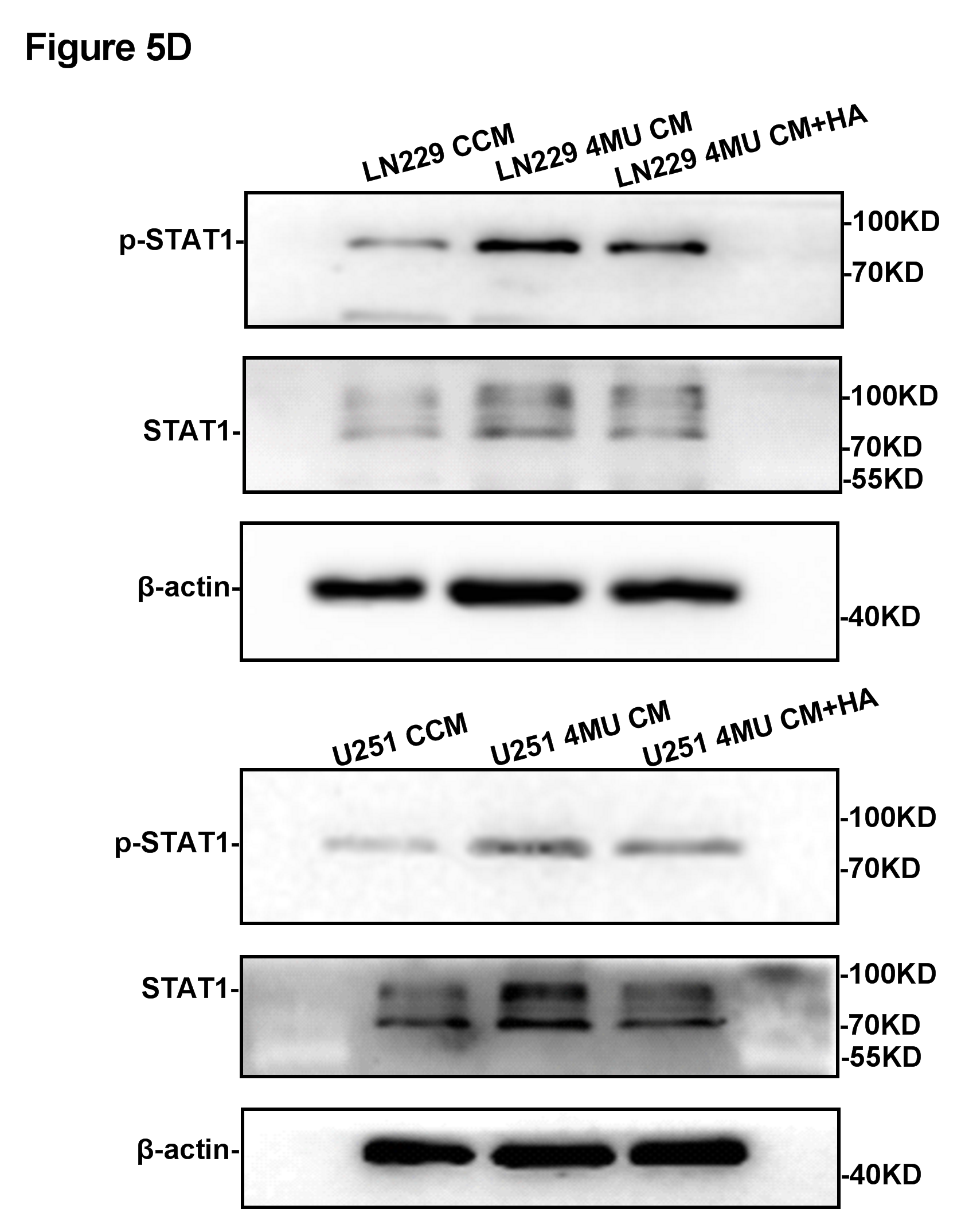


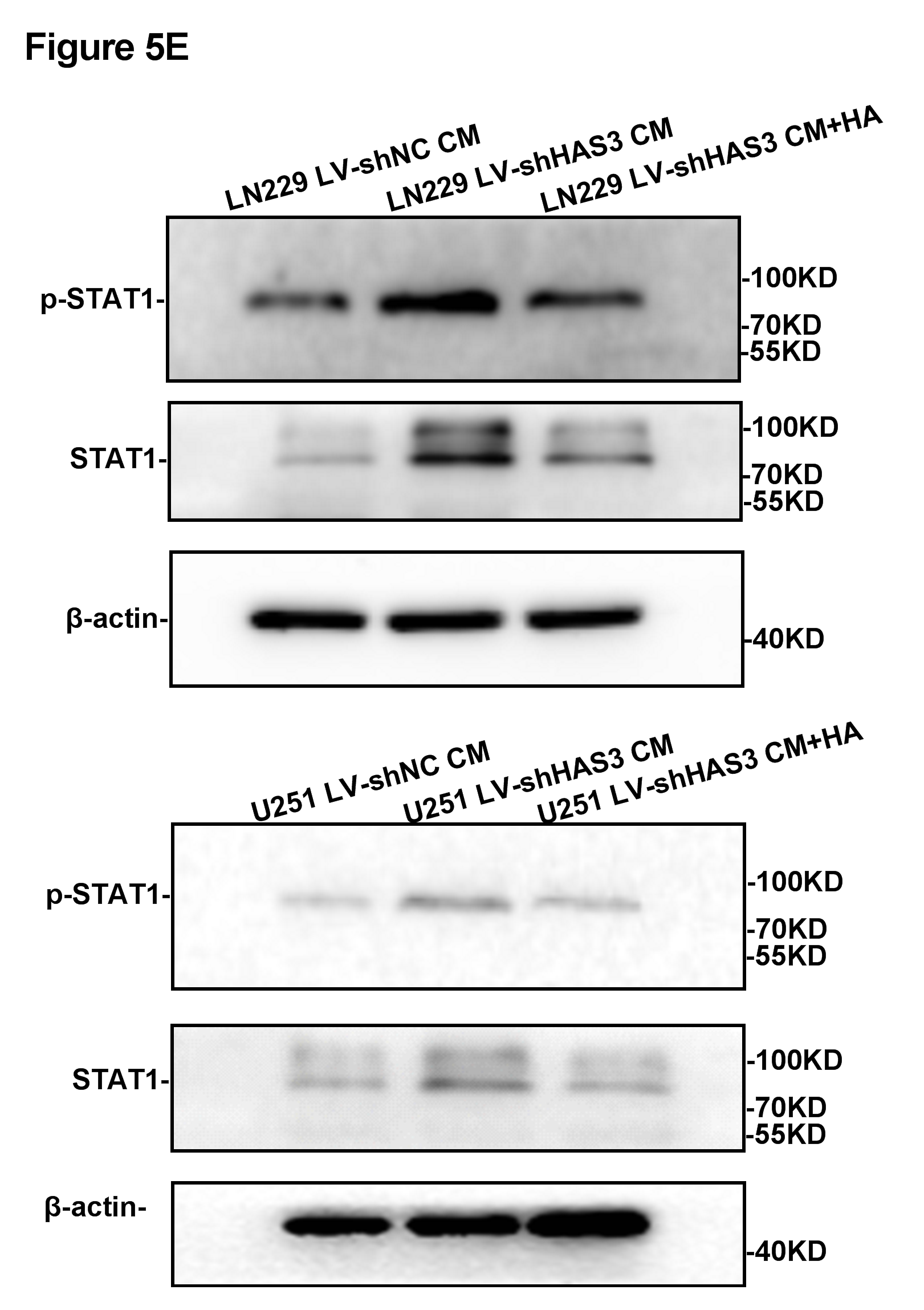


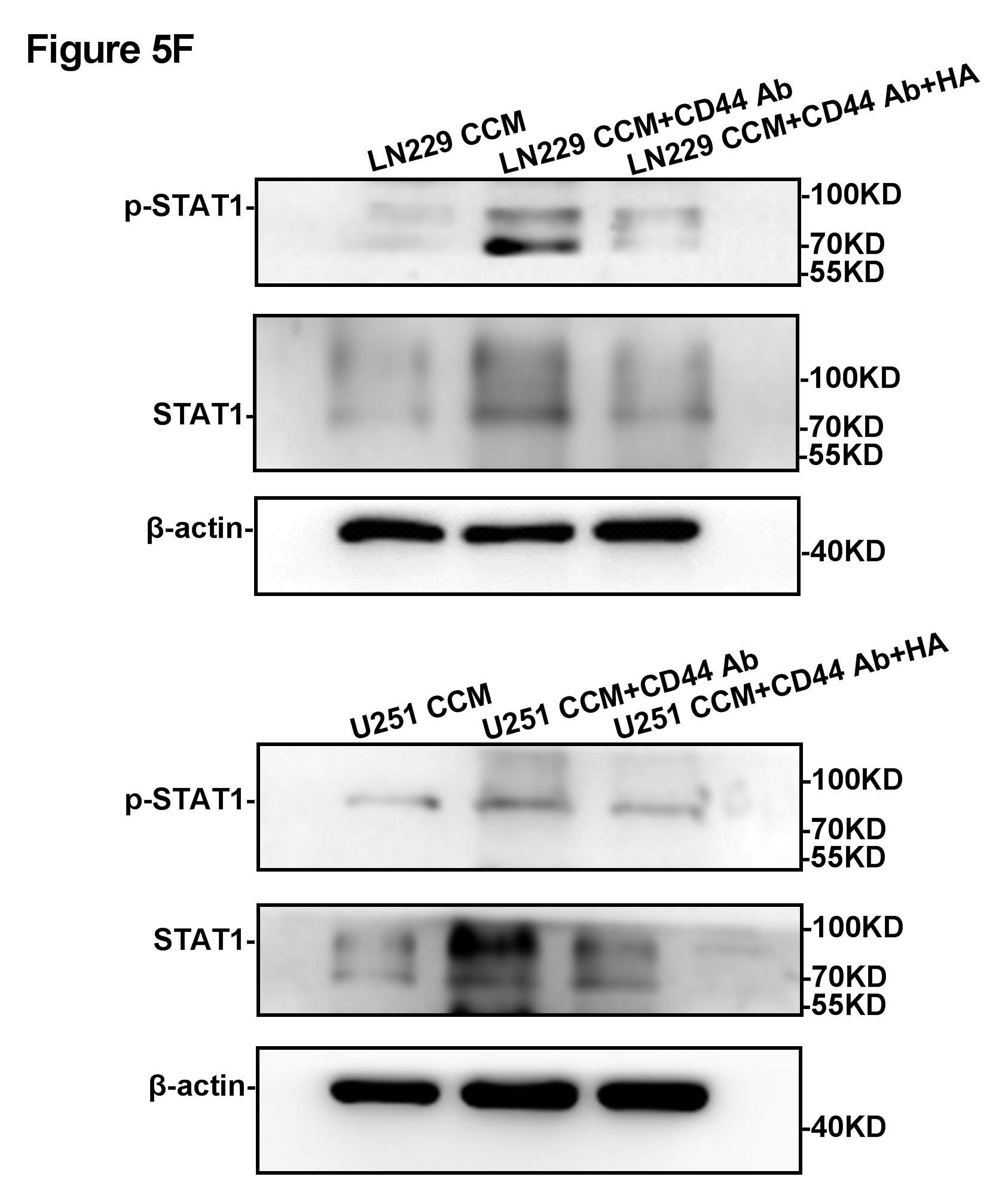


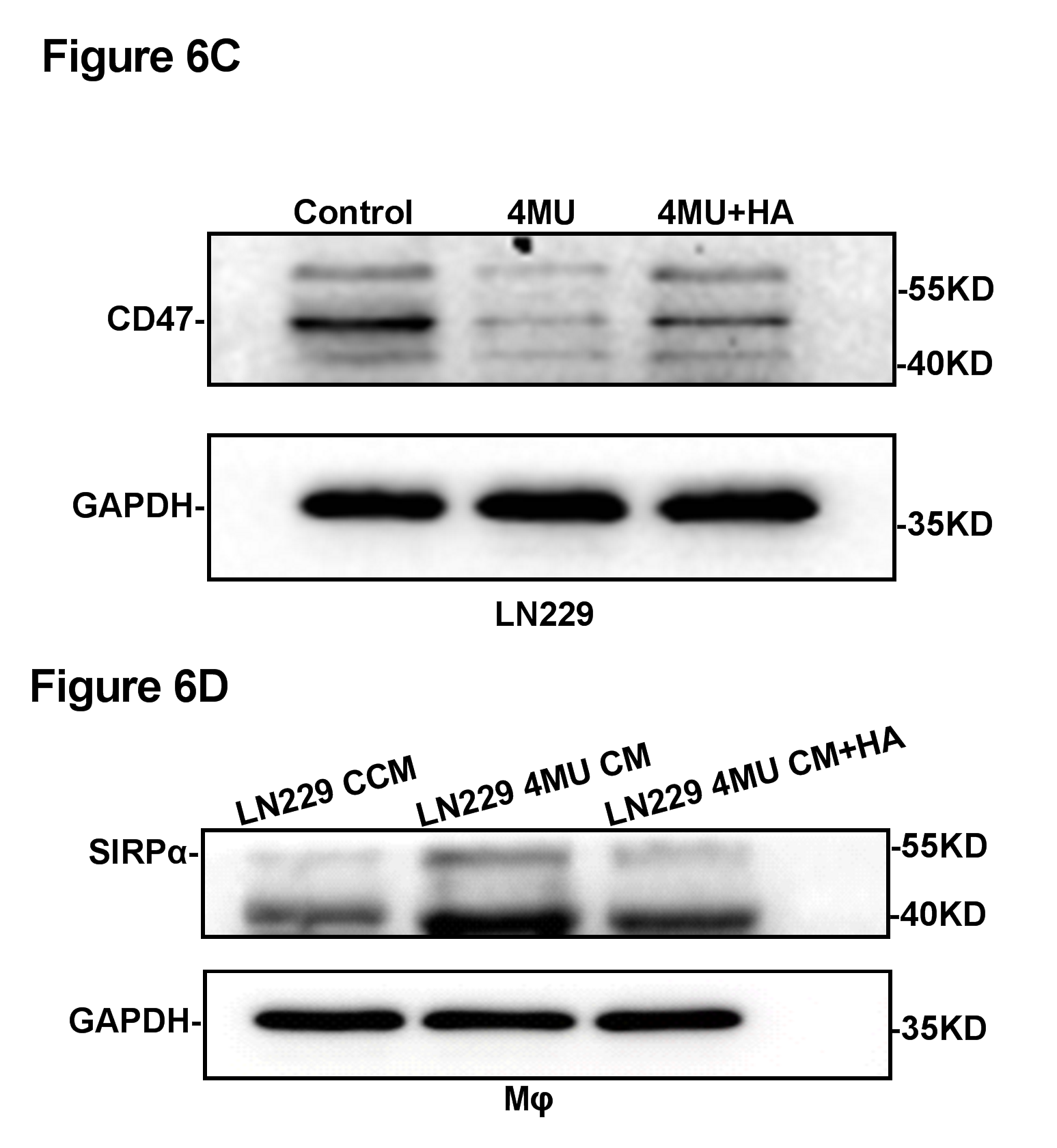


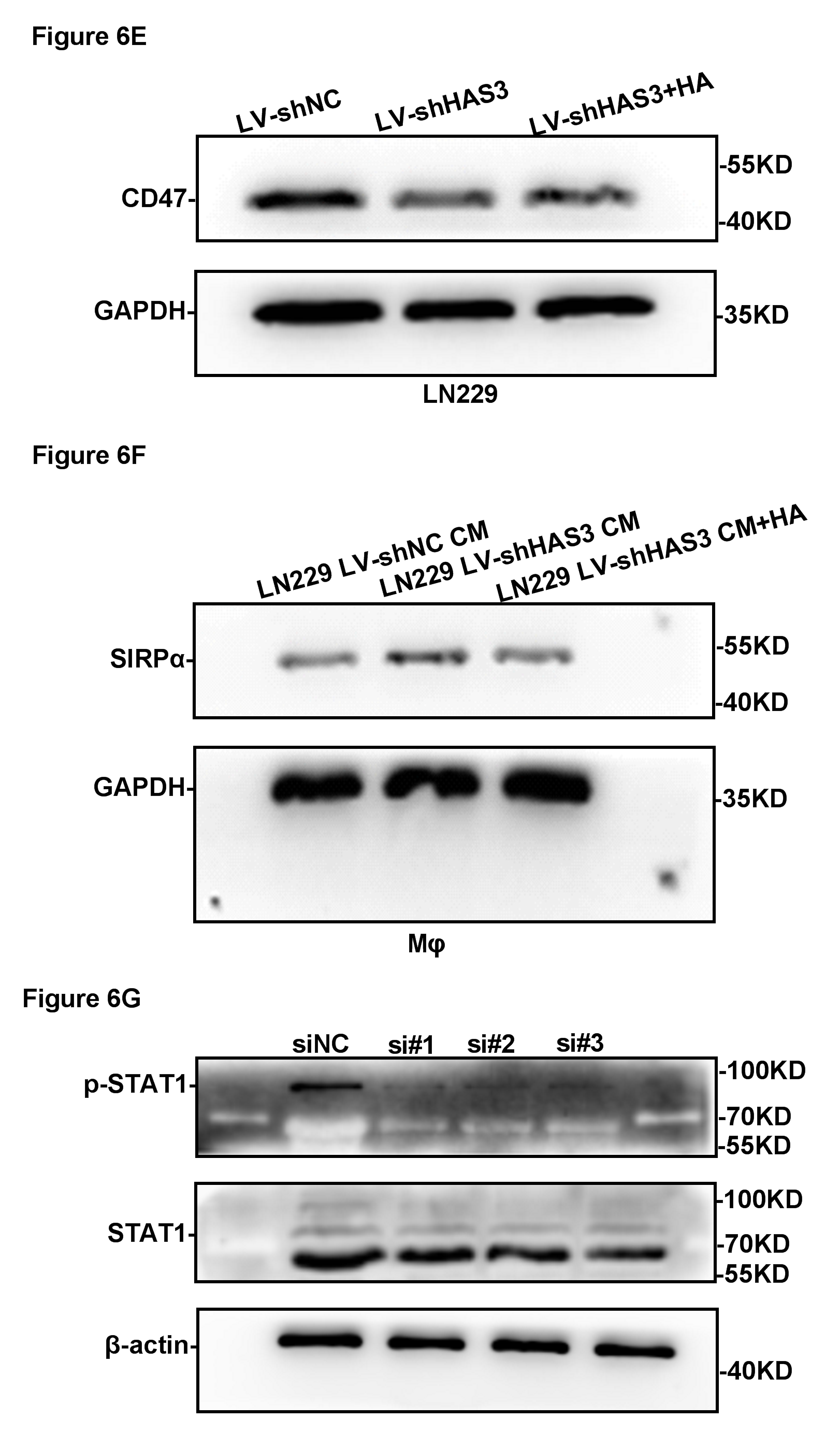


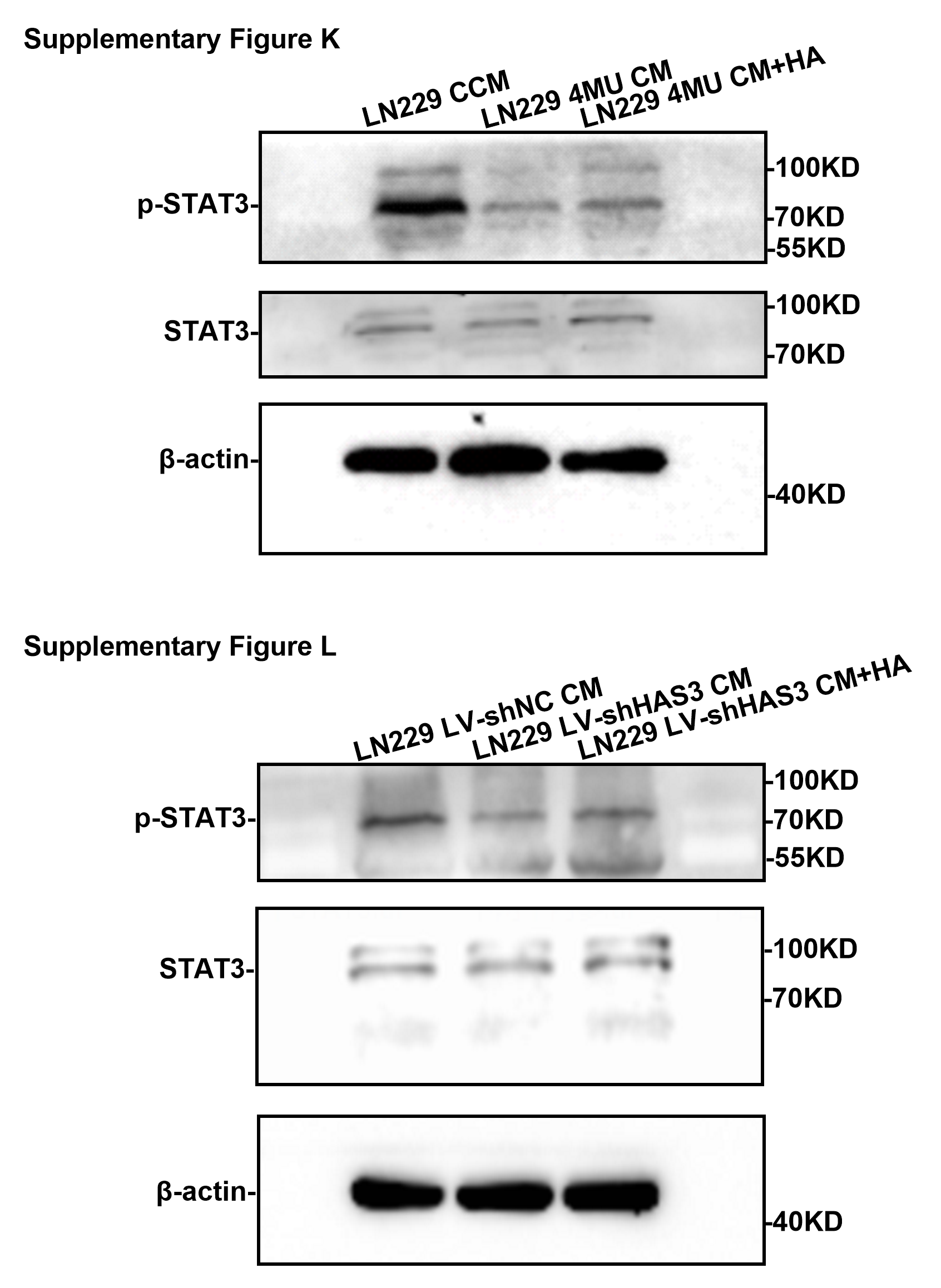


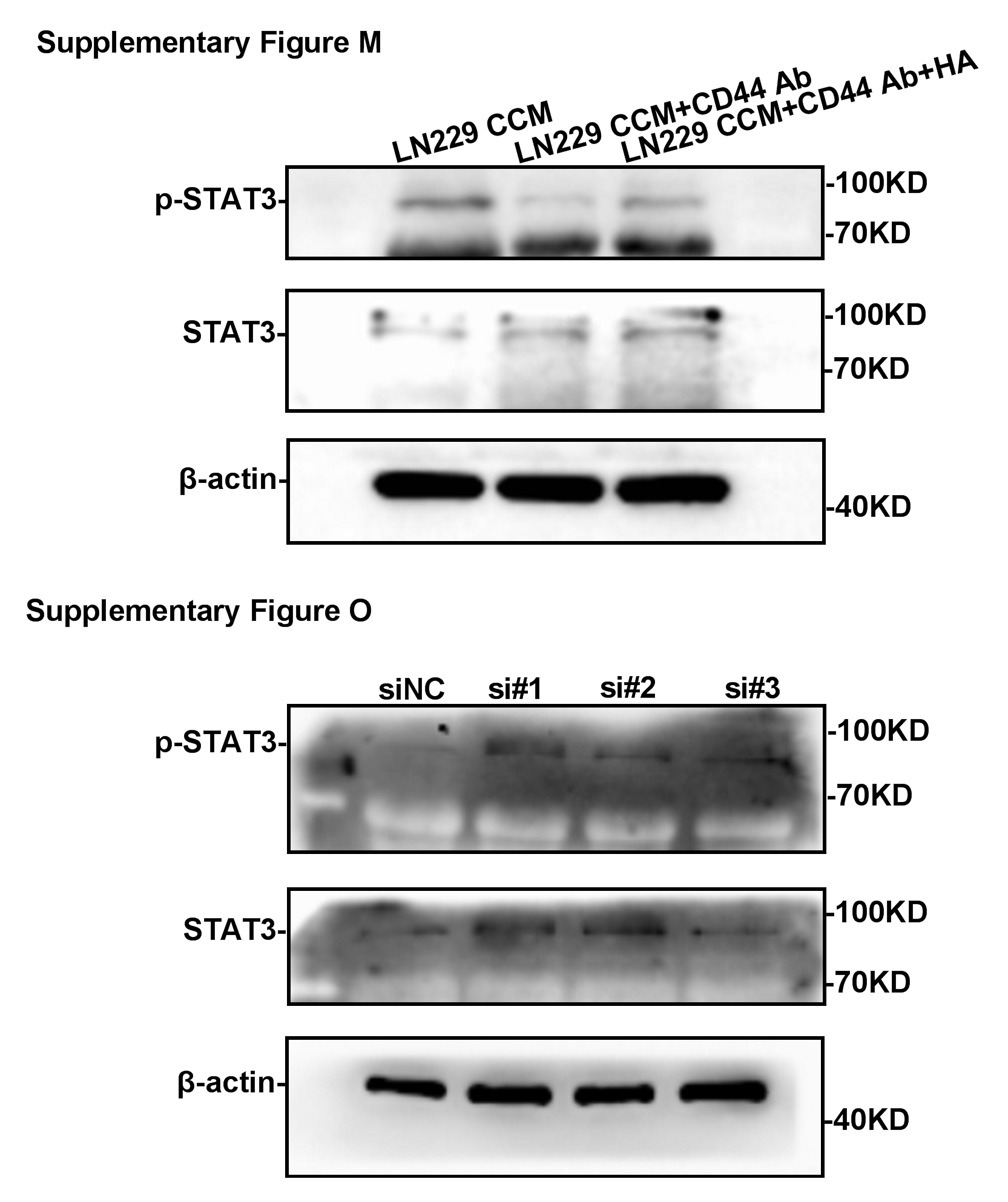

Supplement: Supplementary file 1 — Original western blots [file 41420_2022_973_MOESM1_ESM.docx]

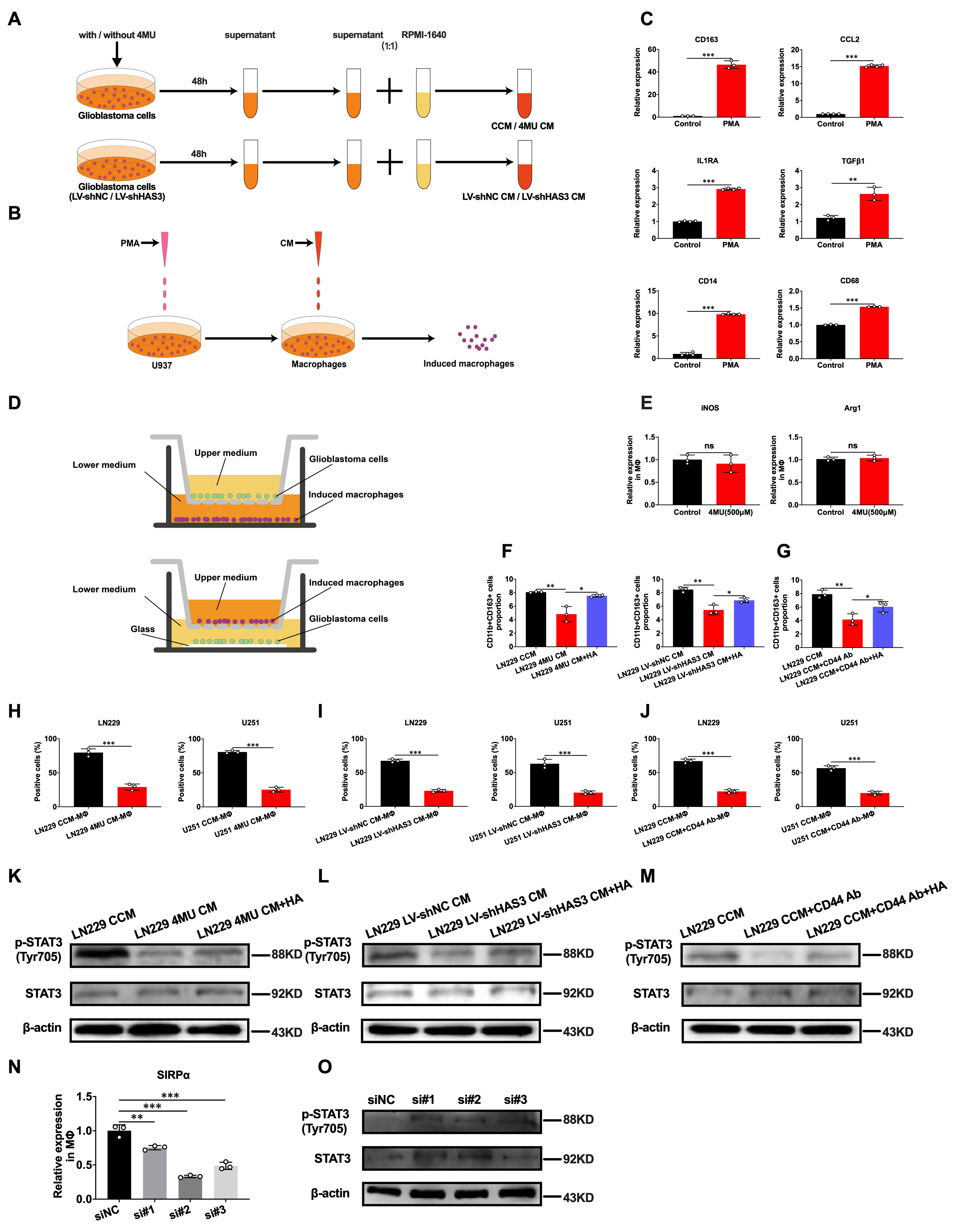

Supplement: Supplementary file 2 — Supplementray Figure [file 41420_2022_973_MOESM2_ESM.png]
