# Supplementary material for: Suppression of the hyaluronic acid pathway induces M1 macrophages polarization via STAT1 in glioblastoma
Source: Cell Death Discov. 2022 Apr 11;8:193. doi: 10.1038/s41420-022-00973-y (PMC9001679; doi:10.1038/s41420-022-00973-y)
Supplement: Supplementary file 3 — Supplementary Figure Legend [file 41420_2022_973_MOESM3_ESM.docx]

**Supplementary Figure 1**

1. Schematic diagram for obtaining conditioned medium.
2. Schematic diagram for obtaining induced macrophages.

C. U937 cells were cultured with PMA (100 ng/ml) for 24 h. The mRNA expression levels of CD163, CCL2, IL1RA, TGFβ1, CD14, and CD68 were detected by qRT-PCR.

D. Up: Schematic diagram for detecting glioblastoma migration and invasion abilities; Down: Schematic diagram for detecting glioblastoma proliferation ability.

E. Macrophages were cultured with 4MU (500 μM) for 48h. The mRNA expression levels of iNOS and Arg1 were detected by qRT-PCR.

F. Macrophages were cocultured with different conditioned medium for 48h, one of the treatment groups was extra treated with exogenous HA (25μg/ml). The proportion of CD11b^+^ CD163^+^ macrophages was detected by flow cytometry.

G. Macrophages pretreated with the anti-CD44 antibody (6μg/ml) were cultured with CCM for 48h, one of the treatment groups was extra treated with exogenous HA (25μg/ml). The proportion of CD11b^+^ CD163^+^ macrophages was detected by flow cytometry.

H-J. The EdU assay results shown in Fig 3A-B, Fig 3C-D, and Fig 4A-B were quantified.

K-M. The protein levels of p-STAT3 and STAT3 were detected by Western blotting in macrophages induced by different conditioned medium.

N. The relative expression level of SIRPα mRNA was detected by qRT-PCR after SIRPα was knocked down in macrophages.

O. The relative protein levels of p-STAT3, STAT3 in macrophages with SIRPα knockdown were determined by Western blotting.

Error bars shown mean ± SD. *P < 0.05, **P < 0.01, and ***P < 0.001.
